# Heterogeneity of trans-callosal structural connectivity and effects on resting state subnetwork integrity may underlie both wanted and unwanted effects of therapeutic corpus callostomy

**DOI:** 10.1016/j.nicl.2016.07.010

**Published:** 2016-07-26

**Authors:** Peter Neal Taylor, Rob Forsyth

**Affiliations:** a Institute of Neuroscience, Newcastle University, UK; b School of Computing Science, Newcastle University, UK; c Institute of Neurology, University College London, UK

**Keywords:** Diffusion weighted imaging, Connectome, Network, Corpus callosum, Epilepsy

## Abstract

**Background:**

The corpus callosum (CC) is the primary structure supporting interhemispheric connectivity in the brain. Partial or complete surgical callosotomy may be performed for the palliation of intractable epilepsy. A variety of disconnection syndromes are recognised after injury to or division of the CC however their mechanisms are poorly understood and their occurrence difficult to predict. We use novel high resolution structural connectivity analyses to demonstrate reasons for this poor predictability.

**Methods:**

Diffusion weighted MRI data from five healthy adult controls was subjected to novel high-resolution structural connectivity analysis. We simulated the effects of CC lesions of varying extents on the integrity of resting state subnetworks (RSNs).

**Results:**

There is substantial between-individual variation in patterns of CC connectivity. However in all individuals termination points of callosal connections mostly involve medial and superior sensory-motor areas. Superior temporal and lateral sensory-motor areas were not involved. Resting state networks showed selective vulnerability to simulated callosotomy of progressively greater anterior to posterior extent. The default mode network was most vulnerable followed by, in decreasing order: frontoparietal, limbic, somatomotor, ventral attention, dorsal attention and visual subnetworks.

**Conclusion:**

Consideration of the selective vulnerability of resting state sub-networks, and of between-individual variability in connectivity patterns, sheds new light on the occurrence of both wanted and unwanted effects of callosotomy. We propose that beneficial effects (seizure reduction) relate to disruption of the default mode network, with unwanted “disconnection syndrome” effects due to disruption particularly of the somatomotor and frontoparietal RSNs. Our results may also explain why disconnection syndromes primary reflect lateralised sensory-motor problems (e.g. of limb movement) rather than midline function (e.g. tongue movement). Marked between-subject variation in callosal connectivity may underlie the poor predictability of effects of callosotomy. High resolution structural connectivity studies of this nature may be useful in pre-surgical planning of therapeutic callosotomy for intractable epilepsy.

## Highlights

• Corpus callosum (CC) damage can result in many, varied effects.
• Using diffusion MRI we show hypothesized termination points of CC connections.
• Unwanted effects such as sensory-motor deficits are predicted.
• We investigate, with selective lesioning, wanted effects for epilepsy surgery.
• Substantial between-subject differences may explain the varied effects of CC injury.

## 1 Introduction

The corpus callosum (CC)^1^ is the major anatomical structure supporting inter-hemispheric connectivity in the brain. It may be congenitally absent (Benezit et al., 2015), or callosal disconnection can be acquired as a result of traumatic brain injury (Basu et al., 2015). In the latter case the disruption is usually partial (Dennis et al., 2015): the splenium of the CC is a characteristic site for diffuse axonal injury as bio-mechanical factors result in concentration of forces at that site. Callosotomy is also performed therapeutically in the amelioration of intractable epilepsy, particularly for atonic drop seizures.

A range of disconnection syndromes have been described following partial or complete callosotomy. These include the supplementary motor area (SMA) syndrome, the anarchic (or alien) hand syndrome, tactile dysnomia, hemispatial neglect, non-dominant hand agraphia and alexia without agraphia (for review see Jea et al. (2008)). The mechanisms of these at times striking and bizarre phenomena are not well understood and their occurrence is poorly predictable. Several of these syndromes can improve spontaneously over time: again the mechanisms of this improvement are unclear.

In previous work we have demonstrated the value of connectomic perspectives on the occurrence and resolution of disconnection syndromes. In one of the youngest reports of anarchic hand syndrome (Basu et al., 2015) we described marked reduction in structural connectivity between homologous superior frontal areas and in functional connectivity between homologous posterior cingulate areas; and hypothesized that restoration of interhemispheric connectivity via trans-cerebellar routes may have contributed to resolution. A number of qualitative analyses of the effects of callosotomy in terms of the anatomy of inter-hemispheric white matter tracts as revealed by Diffusion Tensor Imaging (DTI) have been published (Jang et al., 2013, Le et al., 2005, Molko et al., 2002). In Molko et al.'s case study, loss of transcallosal structural connectivity between homologous areas involved in visual word recognition was associated with corresponding impairments in task-related functional MRI (fMRI) activation and clinical alexia (Molko et al., 2002).

Statistical analysis by techniques such as independent component analysis of patterns of spontaneous fluctuation in brain activation (as revealed at low frequencies by Brain Oxygen Level Dependent (BOLD) signal fMRI) identifies groups of brain areas that tend to activate and de-activate in synchrony known as resting state networks (RSNs). RSN models can be derived at different levels of resolution: typically 7 to 20-network models are generated. Within limits the general configuration of these networks is robust and reproducible (Smith et al., 2009, Yeo et al., 2011). Functional interpretations have been assigned to these RSNs based on the functional activation literature (Yeo et al., 2011) (but see Discussion). In this paper we use novel high-resolution techniques to define the relative vulnerabilities of different RSNs to complete and partial in silico “virtual callosotomies” and relate this to subject-specific consequences (desirable and otherwise) of callosotomy.

## 2 Materials and methods

### 2.1 Imaging data

Two healthy adult control public-domain datasets were used in this study. The older NKI dataset (Nooner et al., 2012) provides repeated scans on the same individual and was used to verify the within-subject reproducibility of our novel high-resolution connectivity pipeline. One T1 weighted MRI image and two separate diffusion weighted MRI images (scan session 1 and scan session 2) are available for one subject. The T1 scanning protocol parameters are as follows: temporal resolution (TR) = 2500 ms, TE = 3.5 ms, inversion time (TI) = 1200 ms, voxel size of 1 mm isotropic. For diffusion acquisition a multiplexed, multiband echo planar imaging sequence was used (Moeller et al. (2010); Xu et al. (2013)). This included acquisition of 128 direction imaging volumes at a b value of 1500 s/mm^2^, along with 9 b = 0 images, TR = 2400 ms, TE = 85 ms with an isotropic voxel size of 2 mm. Further acquisition details are available at http://fcon_1000.projects.nitrc.org/indi/pro/eNKI_RS_ http://fcon_1000.projects.nitrc.org/indi/pro/eNKI_RS_TRT/DIff_137.pdf. This data has been used before for assessing scan-rescan reproducibility of network measures (Zhao et al., 2015).

To investigate between subject differences we used the newer HCP dataset (Glasser et al., 2013) which uses a highly customized protocol. T1 weighted images were acquired at 0.7 mm isovoxel resolution with the following parameters: TR = 2400 ms, TE = 2.14 ms, TI = 1000 ms. For diffusion data, a total of 270 diffusion sampling directions were used in three shells of b values 1000, 2000 and 3000 in addition to 18 b0 volumes at a resolution of 1.25 mm isovoxel. Other parameters were as follows: TR = 5520 ms, TE = 89.5 ms. Full details of the acquisition protocols can be found at http://www.humanconnectome.org/documentation/Q1/imaging-protocols.html. Subject characteristics are included in Supplementary Table 1.

### 2.2 Image processing high resolution pipeline

**Fig. 1:**
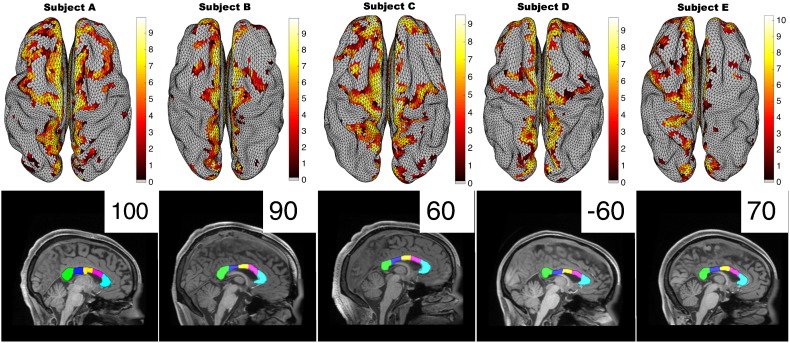
Substantial variations in CC connectivity patterns between subjects. Five randomly selected subjects from the HCP database with excellent CC segmentation (bottom panels). Top panels show termination points of callosal connections in each subject on a grey matter mesh (partially inflated to aid visualisation in sulci). Although the majority of connectivity is para-sagittal in all cases, there is substantial qualitative between-subject variability. Colour coding indicates log(1 + n) where n is the median of the number of tracelines passing through the CC to each of the three vertices of each triangle. The subdivision of the CC into five segments shown in the bottom panels relates to the modelling of partial callosotomy (see Section 3.3). The numbers ranging from 100 to + 100 above the midline coronal images are the Edinburgh Handedness Index (EDI, Oldfield (1971)) score for each individual: − 100 represents a strongly left-handed and + 100 a strongly right-handed individual with ambidextrous individuals scoring zero.

FreeSurfer recon-all was used to generate the cortical surface mesh of NKI data from the T1 image. The white matter surface mesh was then expanded using mris_expand to generate the cortical mid-surface half way between the grey and white matter (Dale et al., 1999, Fischl et al., 1999, Fischl, 2012). The mid-surface was resampled to 16,000 triangles producing the surfaces shown in Fig. 1 using iso2mesh (Fang and Boas, 2009). For HCP data these steps are preprocessed and available to download at https://db.humanconnectome.org/. For HCP data, resampling was precomputed with Caret software (Van Essen et al., 2001).

HCP diffusion data was downloaded preprocessed (Glasser et al., 2013). NKI data was corrected for eddy current distortions and motion using FSL eddy correct using the first b0 image as reference (Jenkinson and Smith, 2001, Jenkinson et al., 2012). Following eddy correction we rotated the b vectors where appropriate using the dt_rotate_bvecs tool. All diffusion data was reconstructed using generalised q-sampling imaging (Yeh et al., 2010) with a diffusion sampling length ratio of 1.25. The diffusion data and the FreeSurfer processed T1 image were then linearly registered to the same space, and the FreeSurfer segmented corpus callosum (Desikan et al., 2006) used as a seed region for tracking. Grey matter regions were combined into one volume region of interest (ROI) and specified as termination and end point criteria. The registration of the grey matter ROI, the CC ROI, and the diffusion MRI was checked manually in all cases for accuracy. The bottom panels in Fig. 1 demonstrate the CC ROI registration quality. The CC was subdivided into five ROIs which were equally spaced along the primary eigendirection using FreeSurfer.

A deterministic fibre tracking algorithm (Yeh et al., 2013) was used, allowing for crossing fibres within voxels. A total of 1,000,000 tracts were computed with lengths between 10 mm and 300 mm. A fixed step Euler algorithm was used for tracking with the step size set to half the voxel size. Anisotropy and angular thresholds were set to 0.6* Otsu's threshold and 60 degrees respectively.

Once tractography was complete, tract end points were saved in the same space as the FreeSurfer processed T1 image. Tract end points and grey matter midsurfaces were loaded into Matlab and registration quality visually confirmed. To generate connectivity profiles (e.g. top panels in Fig. 1) we looped through each endpoint and assigned it to the closest point (shortest Euclidean distance) on the 16,000 triangles comprising the surface mesh. This gives a list of the number of connections for each point of each triangle. To colour the triangles on the surface plot we show the median value of the three triangle points.

The models of functional resting state network connectivity reported by Yeo et al. (2011) were used. The allocation of the 16,000 triangles to the published anatomical cortical surface boundaries of the subnetworks of the 17-network model was performed using preprocessed data from https://db. humanconnectome.org.

## 3 Results

The reproducibility of our high-resolution connectivity method was confirmed using scan-rescan DWI data from a single subject in the NKI dataset (Fig. S1). The scan-rescan correlation of traceline counts through the CC from each high-resolution triangle was high (Spearman's *ρ* = 0.68, *p* < 0.0001). Since tractography uses random initial conditions, we also examined the reproducibility of the tractography and subsequent processing, repeating the entire pipeline using the same data. Reproducibility was excellent (Spearman's *ρ* = 0.97, *p* < 0.0001).

### 3.1 Between-subject variation in connectivity

Between-subject differences in callosal connectivity patterns were examined using the high-resolution HCP data. Five individuals selected at random, with excellent callosal segmentation, were chosen. Fig. 1 shows substantial qualitative between-subject differences in connectivity patterns. For example, subject C has connections involving superior motor areas in both hemispheres, whereas in subject E the connectivity in the left hemisphere is much more dispersed than in the right hemisphere.

**Fig. 2:**
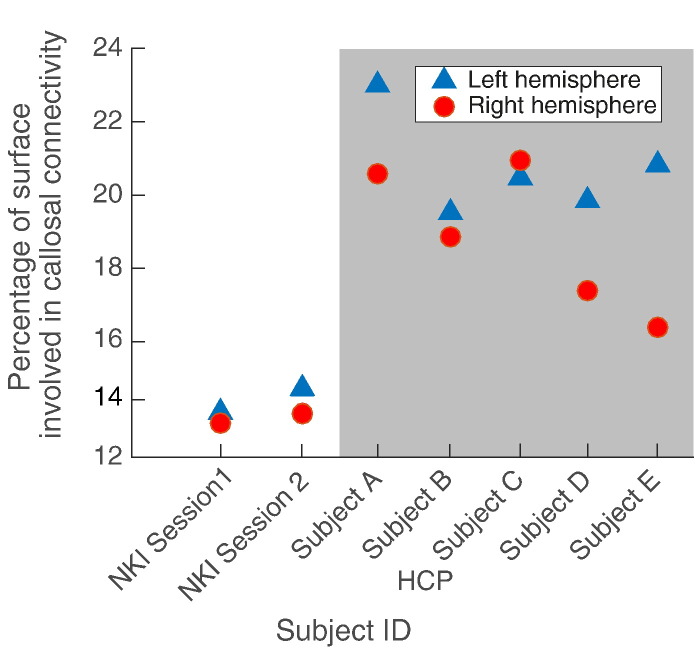
Quantitative differences in callosal connectivity coverage between hemispheres and between subjects. Quantitative confirmation of the heterogeneity shown qualitatively in Fig. 1 with between-subject variability in the proportion of the surface areas of left and right hemispheres connected to the CC (e.g. compare subjects C and E). The reproducibility of our method in the NKI subject is also demonstrated. Triangles and circles represent left and right hemispheres respectively.

This between-subject variability is quantitated in Fig. 2, which shows the proportion of the surface of each hemisphere involved in CC connectivity in the five HCP subjects and the one NKI subject scanned twice. Connectivity of the surface of the left (dominant) hemisphere is generally greater than the right (Fig. 1). The greater proportion of coverage (between 16.4% and 23%) in the HCP data compared to the ≈ 13% of the NKI subject reflects the higher resolution of the HCP data.

### 3.2 Effects on subnetwork integrity

**Fig. 3:**
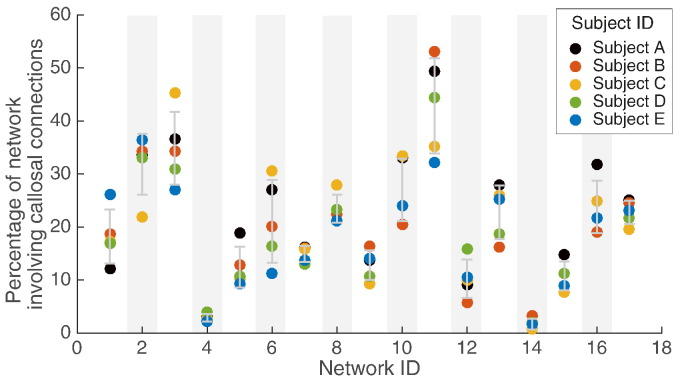
Quantitative differences between subjects, but broad similarities. The percentage of each network involved in callosal connectivity for each subject. Broadly, networks 3 and 11 are most involved in all subjects, with networks 4 and 14 least involved. However, significant variations exist (e.g. network 6, range 11% 31%).

**Fig. 4:**
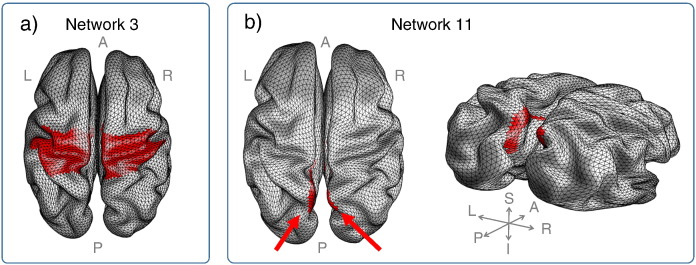
Networks 3 and 11. Surface plots showing the location of Networks 3 and 11 for subject D. Network 3 is involved superior somatomotor function. Network 11 is involved in frontoparietal, default, and dorsal attention network function.

Predicted effects of callosal injury on the integrity of resting state subnetworks were examined by repeating the analyses of Fig. 2 for subsets of the cortical surface for each subject; the boundaries of these subsets being those of the 17-network parcellation defined by Yeo et al. (2011). Fig. 3 demonstrates the involvement of each of the networks in callosal connectivity. Although between-subject variation is again evident there are commonalities: Networks 3 and 11 have high involvement in callosal connectivity and are thus predicted to be particularly vulnerable to complete CC division. Conversely Networks 4 and 14, with very limited involvement in CC connectivity, are predicted to be relatively robust to CC division. The anatomical locations of Networks 3 and 11 are shown in Fig. 4. Network 3 involves superior sensory-motor areas (Fig. 4a) and Network 11 the precuneus (Fig. 4b).

### 3.3 Effects of partial callosotomy

**Fig. 5:**
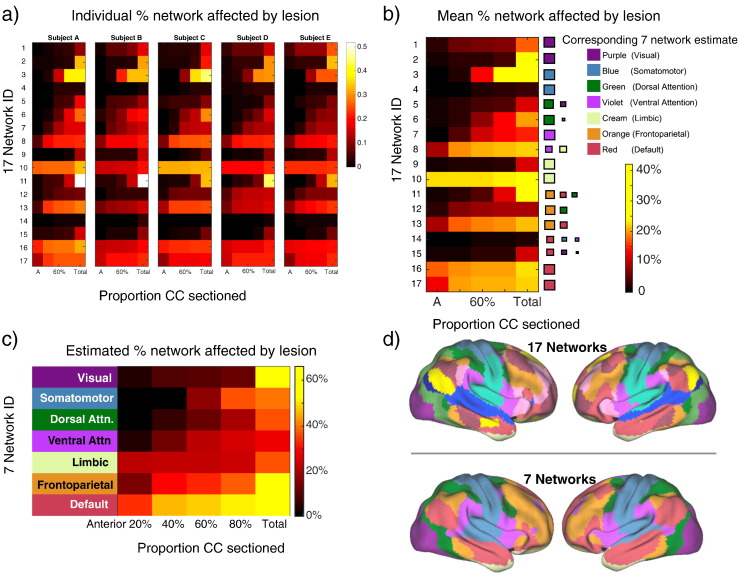
Modelling partial callosotomy. Figures show the proportion of fibres of each resting state network passing through the anterior 20%, 40%, 60%, etc. of the CC, and thus the predicted effects of anterior-fifth, anterior-two-fifths, etc. callosotomy, on the integrity of resting state networks. Panel a shows data for the five individuals shown in Fig. 1. Numbers on the vertical axis are the network numbers from the Yeo 17-network model. The horizontal axis shows progressive divisions (in 20% increments) of the CC from Anterior, through to total simulated resection. Panel b shows the average for the five subjects in panel a. Panel c shows the data of panel b, downsampled to the Yeo 7-network model of functional connectivity for clarity and ease of interpretation. The mapping between the 7 and 17-network models' networks is shown in the legend to panel b: the relative contributions of the *n* = 17 model's networks to each of the *n* = 7 model's networks are indicated by the sizes of the coloured squares. Panel d shows the 7 and 17 networks locations (reproduced from Yeo et al. (2011)). (For interpretation of the references to colour in this figure legend, the reader is referred to the web version of this article.)

Finally the effects of partial callosotomy were modelled. The CC was divided into fifths as indicated in the bottom panel of Fig. 1 for each subject, and the analyses of Fig. 3 repeated in turn for involvement of networks in the anterior 20% of the CC only, the anterior 40%, 60% and 80%. Fig. 5 summarises these findings, individually (Fig. 5a) and averaged across all five HCP subjects (Fig. 5b). The right hand columns of Fig. 5a (i.e. those at total, 100% resection) are essentially the data reproduced from Fig. 3. This demonstrates that although there are differences between subjects for complete resection (Fig. 3), the general trends for partial resection are broadly similar (Fig. 5a).

To aid interpretability, in Fig. 5c the 17-network resolution of Fig. 3 has been downsampled to the Yeo 7-network model of functional connectivity using Supplementary Table 2. These networks are labelled using accepted terminology (but see Discussion). Predictably, the visual resting state network is most robust, only significantly affected by complete CC division that includes the posterior-most portions. Conversely the default mode network (DMN) is affected even by division of the anterior fifth of the CC. Frontoparietal, somatomotor and ventral attention networks show intermediate degrees of vulnerability. Comparison of Fig. 3, Fig. 5 show some differences in vulnerability to partial and total callosotomy. Network 10 for example is markedly affected even by anterior 20% callosotomy with little additional disruption from extension posteriorly (networks 16 and 17 show similar if less striking patterns). This reflects the fact that transcallosal connectivity is less for network 10 than 11 (hence the reduced vulnerability to total callosotomy, Fig. 3) but the large majority of transcallosal connections involving network 10 pass anteriorly (so the effects of anterior 20% division are almost as severe as complete division).

## 4 Discussion

The effects of CC division, whether performed therapeutically or as a result of brain disease, are poorly understood and poorly predictable. We have provided insights into these issues through two important innovations. Although DTI-based structural connectivity studies of the CC have been performed previously (Molko et al., 2002, Lebel et al., 2010, Benezit et al., 2015), the spatial resolution achieved in this study, dividing the cortical surface into over 10^4^ surface ROIs, is unprecedented. The second innovation is to consider the consequences of CC division in terms of resting state subnetwork integrity. Network perspectives on brain function provide important complements to the lesion-based perspectives of classical neurology: in particular acknowledging that focal lesions can disrupt the function of distant areas if important connections to or from that distant area pass through the lesion (compare for example two perspectives on the paradigmatic case of Phineas Gage (Van Horn et al., 2012, Damasio et al., 1994)).

The increased spatial resolution of our study highlights important features of patterns of trans-callosal cortical connectivity including asymmetry, and marked inter-individual variability. Whilst asymmetry of trans-callosal connectivity (at least through the splenium) has been reported previously (Putnam et al., 2010) we believe our demonstration of inter-individual heterogeneity is novel. One technical limitation to be aware of in relation to the examination of effects of partial callosotomy, is that it is recognised that there is some regional variation between sections of the CC in the frequency distribution of axon diameters. Median axon diameters may be smaller in the more anterior segments although the very limited human data available suggests between-subject variation in this picture (Phillips et al., 2015). As a further technical note, it has been shown by some authors that callosal tract reconstruction can vary, dependent on tractography parameters (Bastiani et al., 2012). Indeed, tractography does not show actual white matter tracts, rather streamlines which are inferred to follow the same direction of water flow. It follows that the method of inference will alter the results. Hence caution should be exercised in the interpretation as we do not show a ‘gold standard’ of actual tracts. However, we use the same parameters for all subjects. We also demonstrated good reproducibility for scan-rescan data within the same subject, even using lower resolution single shell data acquired with fewer diffusion directions (Fig. S1) giving confidence to our results. Furthermore, the low number of subjects used here could be considered a limitation of this study, however it has been demonstrated to be sufficient at showing between subject differences (e.g. Fig. 1) and within subject reproducibility (e.g. Fig. S1 and see Besson et al. (2014)).

There has been growing interest in patterns of spontaneous fluctuation in brain activity as reflected by the fMRI BOLD signal in the so-called resting state. Statistical techniques such as independent components analysis can be used to identify brain regions whose patterns of activation and deactivation tend to synchronise and are thus presumed to be functionally linked. The Yeo et al. 7 and 17-subnetwork models of this complex picture are widely accepted (Yeo et al., 2011). Although different groups' models differ in detail the reproducibility and robustness of these models of organisation of brain activity are good (Yeo et al., 2011). Our work predicts that resting state sub-networks (RSNs) will be selectively vulnerable to CC division, and emphasises important between-subject variability in these vulnerability patterns (see Fig. 3). Both these factors may be pertinent to the challenge of predicting both wanted and unwanted effects of CC division.

Our demonstration of a parasagittal location for cortical regions with significant trans-callosal connections (1) is consistent with prior literature emphasising connections between homologous Supplementary Motor Areas (SMAs) in the CC (Fling et al., 2013). Fig. 3 predicts that Networks 3 and 11 are most vulnerable to complete division of the CC, with Networks 4 and 14 predicted to be most robust. Clinical interpretation of these predictions is aided by down-sampling the Yeo 17-network model data to the 7-network model (compare Fig. 5b and c). The former is approximately, although not exactly, a superset of the latter: for example the “Visual” network of the 7-network model is divided into Networks 1 and 2 of the 17-network model; and Networks 9 and 10 are subdivisions of the “Limbic” network of the 7-network model. The namings of the 7-network model RSNs (“default”, “somatomotor”, etc.) are widely used but recognised to be oversimplifications (Yeo et al., 2011). In terms of the vulnerable networks of Fig. 3, Network 3 comprises the medial part of the Somatomotor network of the 7-network model; Network 11 maps particularly to the Frontoparietal and to some extent Default Mode networks. Conversely the two robust networks of Fig. 3, Networks 4 and 14, map (respectively) to the more lateral portion of the 7-network model Somatomotor network and to the Default Mode networks. The differential vulnerability of Networks 3 and 4 the medial and lateral portions respectively of the Somatomotor network of the 7-network model reflects the parasagittal distribution of cortical regions with significant trans-callosal connections demonstrated in Fig. 1. Network 3 corresponds in large part to the more medially placed Supplementary Motor Area (SMA): this is consistent with the well-described post-callosotomy “SMA syndrome” comprising paresis of the non-dominant leg, incontinence, reduced speech and or non-dominant hand apraxia. This can occur transiently or more persistently particularly after single-stage complete CC transection (Jea et al., 2008). Trans-callosal connectivity between SMAs was striking reduced in our recently reported case of a child with anarchic hand syndrome (Basu et al., 2015). The CC is not the only structure supporting inter-hemispheric connectivity, which can also happen via the hippocampi, various brainstem level structures and the cerebellum. Delayed re-establishment of communication via such routes may be underlie both resolution of disconnection syndrome symptoms (Basu et al., 2015) and unwanted late recurrence of seizures (Jea et al., 2008).

Fig. 5 indicates that the Default Mode network (DMN) will be selectively affected even by division of the anterior 20% of the CC. At the 17-network model resolution it can be seen that this specifically reflects vulnerability to anterior callosotomy of Networks 17, 16 and to a lesser extent 13. As would be expected these networks particularly involve anterior, parasagittal cortical areas. This may explain why partial anterior callosotomy can be effective in seizure control whilst avoiding the morbidity (including occurrence of disconnection syndromes) associated with more complete sections, including the mid-body of the CC (Geschwind et al., 1995) or the splenium (Kawai et al., 2004). The role of the CC in permitting bilateral synchronisation of spike-wave discharges has been shown in feline (Musgrave and Gloor, 1980) and rodent (Vergnes et al., 1989) epilepsy models. After CC division seizure activity in each hemisphere becomes independent (Musgrave and Gloor, 1980). Seizure propagation through the DMN has been reported as a feature of seizures associated with loss of consciousness in humans, including complex partial, generalised tonic-clonic and absence seizures (Song et al., 2011, Danielson et al., 2011, Jackson, 2014). Thus anterior partial callosotomy can selectively disrupt the network most associated with aggressive seizure propagation. The main indication for partial or complete therapeutic callosotomy is palliation of severe atonic seizures. In a fascinating case report Pizoli et al. (2011) describe restoration of more normal patterns of resting state fMRI BOLD activity after an anterior two-thirds callosotomy in a five year old boy for severe, intractable polymorphic epilepsy including atonic seizures (Lennox Gastaut syndrome). This underlines the dynamic, plastic nature of resting state networks; and also illustrates why the consequences of congenital agenesis of the CC (Owen et al., 2013) differ from those of CC division in later life (after interhemispheric connections have been established). Disconnection syndromes appear to require a degree of prior maturation of callosal structure (Basu et al., 2015) and thus the risk-benefit ratio of complete callostomy may be different at younger ages although delayed onset of anarchic hand symptoms eight years after CC involvement in a middle cerebral artery stroke at the age of three has been reported (Soman et al., 2009). Pizoli et al.'s report also underlines the need to bear in mind that nearly all clinical studies of the effects of callosotomy are in people with pre-existing, severe epilepsy.

An important limitation of this work is that even at the exceptional anatomical resolution of our 16,000 node parcellation, we can only realistically model complete division of parts of the CC. Although callosal injury is commonly seen in the context of traumatic brain injury, histological studies suggest that < 1% CC fibres would typically be involved even in severe diffuse axonal injury and our model does not have the resolution to realistically simulate such partial degradation of CC connectivity (Tasker et al., 2010). For surgical planning however we would suggest accurate localisation of tracts, (e.g. using track density imaging Calamante et al. (2010) rather than arbitrary subdivisions) in the CC patient-specifically should be performed.

The findings of Fig. 5 corroborate in general terms clinical observations (e.g. Kawai et al. (2004)) that where callostomy is being considered, a partial (anterior) division may be preferable, as disconnection syndromes may be more common after complete CC division (Jea et al., 2008). Specifically we predict that disruption of Network 3 predisposes to the occurrence of the SMA and Anarchic Hand disconnection syndromes. The finding of substantial between-subject differences in the specifics of connectivity strongly suggests that high-resolution structural connectivity imaging and prediction of patterns of disruption to resting state subnetworks may be an important part of pre-operative planning for callosotomy. Moving forward, validation of our predictions in a clinical cohort will be necessary.

The following are the supplementary data related to this article.

Fig. S1 Reproducibility of connectivity is high within a subject. Scan-rescan data from NKI database. Termination points of callosal connections plotted on grey matter surface using data from scanning session 1 (a) and scanning session 2 (b) in the same subject show good qualitative agreement in their spatial profile. For orientation, anterior cortical areas are at the top and left is left in the upper plots. Lower plots show a posterior view. c) Quantitative similarity between the two scanning sessions is high (correlation 0.68). d) Quantitative similarity within a scanning session re-running the processing pipeline is excellent (correlation 0.97). Grey lines in c) and d) show least squares regression line of best fit. Fig. S1

Fig. S2 The 17 network parcellation from Yeo et al., (2011). Fig. S2

Table S1 Subject characteristics. Image 1

Table S2 Approximate mappings between the 7 and 17 area Yeo et al. (2011) networks. Table S2

## Footnotes

1 Abbreviations: CC = corpus callosum; fMRI = functional MRI; rs-fMRI = resting state functional MRI; RSN = resting state (sub)network
