# Supplementary figures and images for: Heterogeneity of trans-callosal structural connectivity and effects on resting state subnetwork integrity may underlie both wanted and unwanted effects of therapeutic corpus callostomy
Source: Neuroimage Clin. 2016 Jul 26;12:341–7. doi: 10.1016/j.nicl.2016.07.010 (PMC4983151; doi:10.1016/j.nicl.2016.07.010)

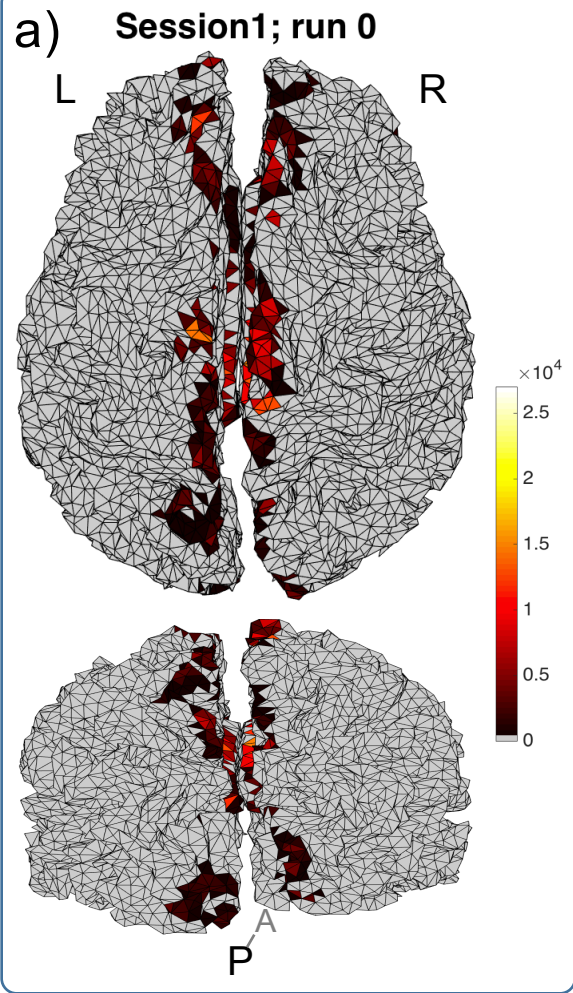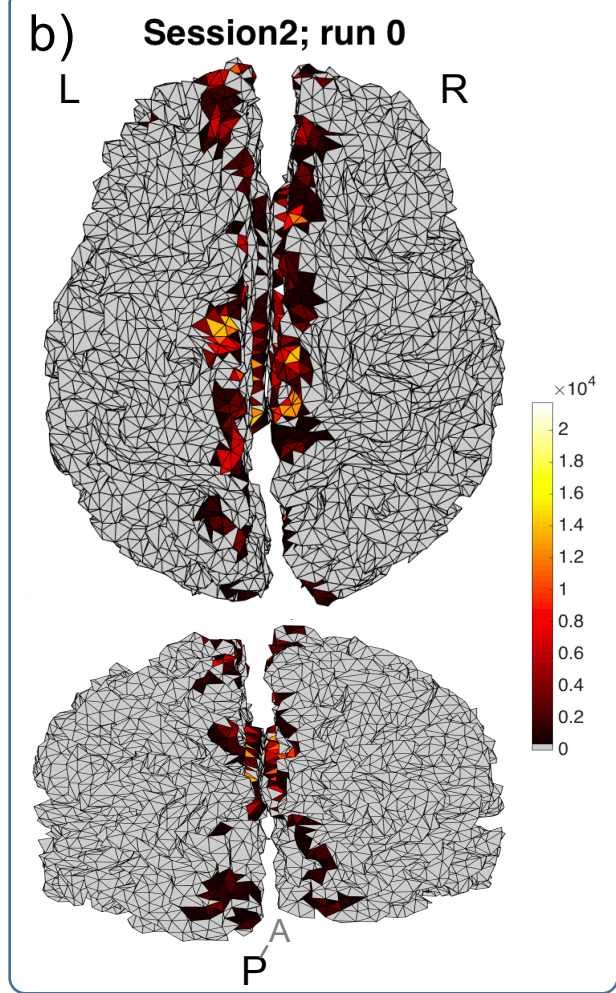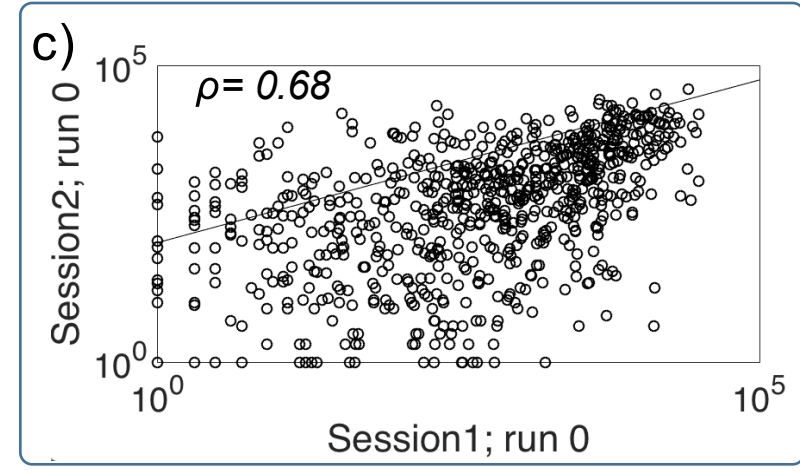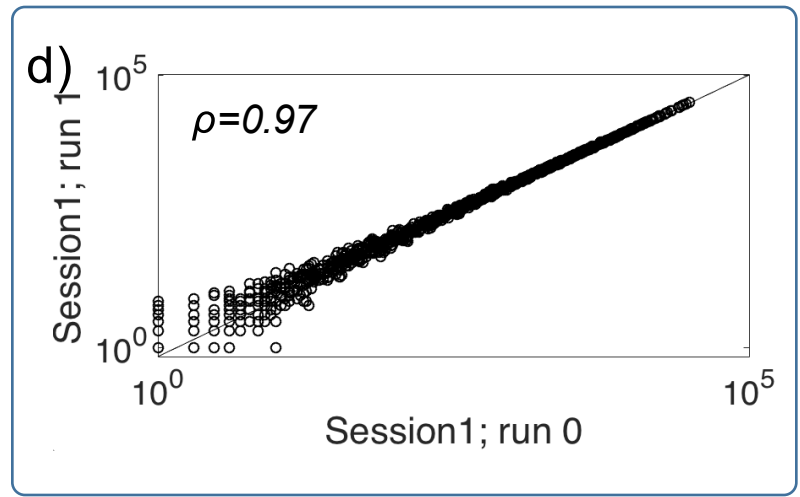

Supplement: Fig. S1 — Reproducibility of connectivity is high within a subject. Scan-rescan data from NKI database. Termination points of callosal connections plotted on grey matter surface using data from scanning session 1 (a) and scanning session 2 (b) in the same subject show good qualitative agreement in their spatial profile. For orientation, anterior cortical areas are at the top and left is left in the upper plots. Lower plots show a posterior view. c) Quantitative similarity between the two scanning sessions is high (correlation 0.68). d) Quantitative similarity within a scanning session re-running the processing pipeline is excellent (correlation 0.97). Grey lines in c) and d) show least squares regression line of best fit. [file mmc1.pdf]

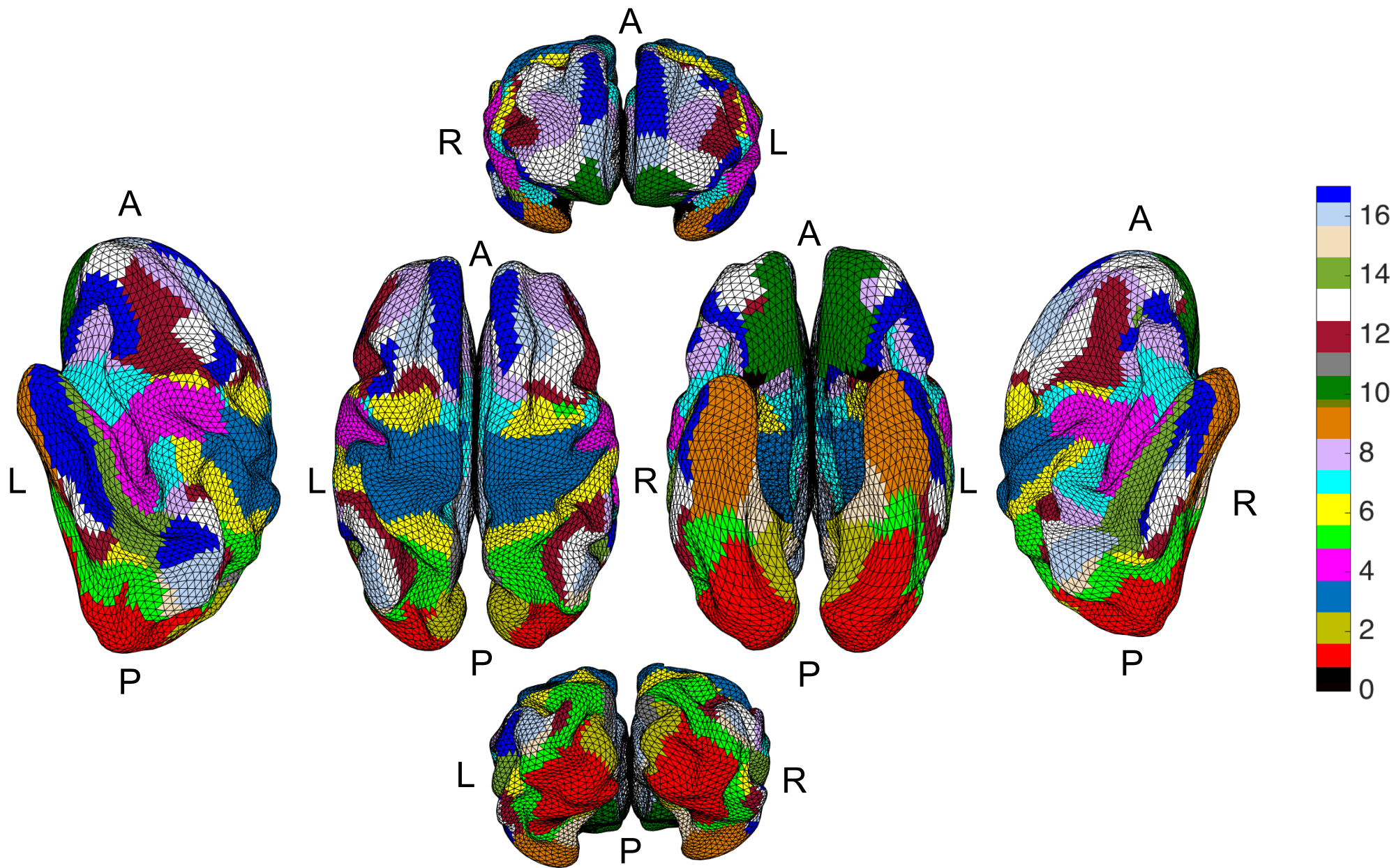

Supplement: Fig. S2 — The 17 network parcellation from Yeo et al., (2011). [file mmc2.pdf]
